# From the Patient's Point of View: A Nurse's Experience as a Patient

**Published:** 1914-11-21

**Authors:** 


### FROM THE PATIENT'S POINT OF VIEW.

### A Nurse's Experience as a Patient.

I have three times had to become a patient in hospital for operations, and, though a nurse, I do not think I was exacting, but perhaps saw more clearly than others when reform was needed.

In two instances I entered the same London hospital, the same ward each time. The surgical treatment I received was excellent, but the nursing left much to be desired. The first night I heard the staff nurse telling the night nurse "No. 13 was a new patient, another nurse, and she was sick of nurses." I had no sleep at all the first night, as my bed was facing the kitchen door, which was left wide open, with the light streaming directly on my face, and the night nurses constantly resorted thither for audible chatter. The second night I was given chloral, and hoped for some sleep; but the day nurse started my preparation for operation before she went off duty at 9 p.m., and left it for the night nurse to finish when convenient, which seemed to be about 1 a.m. Three enemas during the night at two-hourly intervals did not add to the possibility of sleep. The night nurse used to go from one patient to another with the same Higginson's syringe without any sterilising, and thought screens for douching, enemas, etc., quite superfluous.

The second operation I had was a serious abdominal one. I am sure my convalescence was prolonged for want of better nursing during the acute stage. After the operation I was allowed to wait twenty-six hours before the catheter was passed, and when the house surgeon inquired if warm sponging had been tried, barley water given, etc., the nurse replied that everything had been done, whereas nothing had been tried at all, except to leave me on the bed-pan for half an hour at a time, though I was in great pain from a distended bladder.

On the first morning, though flat on my back, I was given the brush and comb and left to do my own hair, and struggle into my dressing-jacket the best way I could. When I wanted anything out of the locker or from the foot of the bed, where the nurses had a knack of putting things just beyond my reach, I had to squirm round till I could reach it, as the nurses were always so rushed. What wonder the wound did not heal completely by first intention, but had to be strapped! One dear little night nurse was most attentive, but, unfortunately, she was only there a short time. The food was good, but served unappetisingly. Sister gave me my choice whenever she could, but no one troubled whether I ate it or not. I was discharged "to make room" on the nineteenth day, having been on my feet the two previous days. I packed my own bag, and struggled down the ward with it when I left. The staff were always kind to me, and said I was a very good patient. The fault, I think, lay in the sister's lack of supervision of the nurses and their training.

A friend of mine had been in the same ward, and she had the same faults to find; and in her time the staff nurse gave round the medicines without washing the glass in between! On two occasions she was given enemas in the vagina.

My third experience was a much happier one when I entered a small special hospital in the provinces for another operation. The nursing was excellent; the only fault I had to find was the large meals I was forced to eat, which I found very disagreeable, as I am naturally a small eater.

I think if all nurses could become patients at some part of their training their patients would greatly benefit.

